# Blastic Plasmacytoid Dendritic Cell Neoplasm: A Comprehensive Review of the Disease, Central Nervous System Presentations, and Treatment Strategies

**DOI:** 10.3390/cells13030243

**Published:** 2024-01-28

**Authors:** Shefali Mehra, Justin Taylor

**Affiliations:** Sylvester Comprehensive Cancer Center, Miller School of Medicine, University of Miami, Miami, FL 33136, USA; shefalimehra@med.miami.edu

**Keywords:** BPDCN, CNS involvement, myelodysplastic syndromes, treatments, tagraxofusp, intrathecal therapy, emerging therapeutics

## Abstract

Blastic plasmacytoid dendritic cell neoplasm (BPDCN) is a rare, aggressive hematologic malignancy with poor outcomes. The World Health Organization (WHO) redefined BDCN as a distinct disease entity in 2016. BPDCN arises from plasmacytoid dendritic cells, manifesting primarily in the skin, bone marrow, and lymph nodes, occasionally involving the central nervous system (CNS). This presents challenges in diagnosis and treatment, with CNS involvement often overlooked in standard diagnostic workups due to BPDCN’s rarity and patients often being neurologically asymptomatic at diagnosis. CNS involvement typically emerges during relapse, yet clinical trials often exclude such cases, limiting our understanding of its development and treatment. Treatment options for CNS involvement include intrathecal (IT) chemotherapies like methotrexate and cytarabine, often in combination with systemic agents. Tagraxofusp and traditional regimens for acute myeloid leukemia show limited success at preventing CNS relapse, prompting exploration of combined therapies like hyperfractionated cyclophosphamide, vincristine, doxorubicin, and dexamethasone (HyperCVAD) with venetoclax and adding IT chemotherapy to other backbones. Ongoing clinical trials investigating emerging therapies offer hope despite limited focus on CNS implications. Trials incorporating CNS-involved patients aim to pioneer novel treatment approaches, potentially reshaping BPDCN management. Understanding CNS involvement’s complexities in BPDCN remains crucial for tailored treatments and better patient outcomes.

## 1. Introduction

Blastic plasmacytoid dendritic cell neoplasm (BPDCN) is a rare and aggressive hematologic malignancy primarily afflicting middle-aged to older men and has poor survival outcomes [1,2]. Although the median age of diagnosis is around 65 years of age, BPDCN has also been seen in younger populations, including children in a bimodal distribution pattern. However, the disease is an exceptionally rare malignancy with the incidence of BPDCN accounting for less than 0.5% of all hematologic malignancies [3,4].

Due to its extremely sparse occurrence, BPDCN has been referred to by many different names. The disease was initially recognized in 1994 as a CD4+ natural killer (NK) cell leukemia and quickly became known as a blastic NK cell lymphoma due to its blastic appearance as well as expression of CD4, CD56, and CD15. The nomenclature of the disease underwent many modifications due to the uncertainty regarding the origin of the cells for this malignancy. However, once the disease was determined to originate from plasmacytoid dendritic cells (pDCs), the World Health Organization (WHO) reclassified the disease in 2008 as a subtype of acute myeloid leukemia (AML) or that of acute lymphoblastic leukemia (ALL). Only in 2016 did the WHO reclassify the disease into a distinct category of a dendritic cell-related disease [5,6,7]. However, in 2022, the WHO put BPDCN in a category labeled “plasmacytoid dendritic cell neoplasms”. Mature plasmacytoid dendritic cell proliferation (MPDC) is now the other disease which exists in this category along with BPDCN. While both conditions have origins with proliferation of pDCs, MPDC is characterized by findings of normal morphologic and mature pDC proliferation, while BPDCN differs with regard to proliferation of the immature pDCs or blasts. It should be noted that MPDC, a premalignant condition, has the potential to become malignant in the form of BPDCN or another kind of myeloid malignancy [8].

The etiology of the disease remains to be determined, as there is a lack of understanding regarding environmental or hereditary factors which contribute to the disease. However, patients with prior hematologic malignancy have been diagnosed with BPDCN. The Gruppo Italiano Malattie Ematologiche dell’Adulto (GIMEMA) study recorded four patients with secondary BPDCN [2]. The Hellenic Dendritic Cell Leukaemia Study Group reported two cases of secondary BPDCN [9]. Patients have been reported to have been initially diagnosed with myelodysplastic syndrome (MDS), AML, and chronic myeloid leukemia. Amongst studies reporting cases of BPDCN patients with prior diagnoses of hematologic malignancies, MDS has been the most common amongst this patient population. However, the relationship between BPDCN and prior melogenic or malignancies is in its initial stages of exploration. For example, Hu et al. demonstrated that BPDCN may also be associated with chronic myelomonocytic leukemia (CMML), with CMML possibly able to transform into BPDCN [10].

BPDCN originates from precursors of pDCs, a subset of dendritic cells primarily involved in immune regulation [11,12]. These malignant cells accumulate in the bone marrow, skin, and lymph nodes, but can also infiltrate other organs like the spleen and peripheral blood [13]. Clinically, BPDCN typically presents as brownish or violaceous cutaneous lesions, appearing as nodules or patches, and may resemble leukemia cutis or myeloid sarcoma [13,14,15,16]. They may also be associated with hyperpigmentation and erythema. Typically, skin biopsies demonstrate a nodular-diffuse dermal infiltration with malignant cells and scant involvement of the epidermis. Though rare, not all manifestations of the disease occur primarily with skin, as some cases of BPDCN have occurred with patients presenting directly with bone marrow infiltration [17]. Symptoms of bone marrow disease includes thrombocytopenia, anemia, leukoneutropenia, and leukocytosis [3]. While skin, bone marrow, and lymph nodes are the primary sites of involvement, it is noteworthy that central nervous system (CNS) infiltration can happen; however, it is a rarer presentation of BPDCN. CNS involvement in BPDCN can result in neurological symptoms and poses additional challenges in diagnosis and treatment [18,19,20]. With the true incidence of CNS presentation in BPDCN being unknown and the CNS possibly being a sanctuary site for the malignancy, studying and shining light on this aspect of the malignancy is imperative. In the article, we aim to review and provide a comprehensive overview of BPDCN with respect to CNS presentations and treatment strategies.

## 2. Pathophysiology of CNS Involvement in BPDCN

BPDCN exhibits intricate genetic and pathogenic features that distinguish it from other malignancies. pDCs are largely responsible for viral defense, as upon maturation, these cells are responsible for secreting cytokines such as type I interferon (IFN), interleukin-6, interleukin-8, and tumor necrosis alpha. This allows for the activation of T-cells, macrophages, and NK cells [1]. Initially, BPDCN was thought to be an NK cell malignancy due to presence of CD4+ and CD56+ cells when immunotyping of cells was conducted. What has proven to be historically difficult with characterizing the disease is that, as has been documented, blasts may not express CD4 or CD56. It was proposed that various co-expressions of certain combinations of cell markers should be looked at in order to make a reliable diagnosis of the disease through immunohistochemistry. Garnache-Ottou et al. proposed that the following co-expression combinations would mean a diagnosis of BPDCN could be made: CD4+, CD56+/−, CD123+ cells, and BDCA-2/4+, and the absence of CD3+, CD11c+, MPO+, and CD79a+ [21]. Other cell surface markers expressed by BPDCN cells include CD303, CD304, T-cell leukemia/lymphoma 1 (TCL1), and Transcription Factor 4 (TCF4) [12]. Studies by Julia et al. and Facchetti et al. demonstrated that having CD4+, CD 56+, CD123+, and TCL-1-expressing cells, would prove to be reliable when making the diagnosis of BPDCN [17,22]. In 2022, the WHO specified that to make the diagnosis of BPDCN, the expression of CD123, CD4, CD56, and another pDC marker (CD303, 304, TCL1, or TCF4) is required [8,23]. In addition to the 2022 WHO guidelines, the North American Blastic Plasmacytoid Dendritic Cell Neoplasm Consortium set guidelines in 2023 recommending testing for TCL-1, TCF4, and CD303 in addition to CD123, CD4, and CD56 [24].

With regard to possible origin of the disease in CNS cells, Sapienza et al. analyzed 51 BPDCN samples which revealed that significant dysregulation in microRNAs may exert a profound impact on neurogenesis. Notably, the neurogenic process was markedly enriched in BPDCN samples compared to normal pDCs, showcasing a distinct pathogenic trait. Genes such as Neuroligin-4X (*NLGN4X*), involved in the development and maintenance of synapse functioning, and neural protein or enzyme markers such as Doublecortin (*DCX*) and Ubiquitin C-Terminal Hydrolase-L1 (*UCHL-1*) were expressed in tumor microenvironments which serve to emphasize the neural involvement in BPDCN [6,25]. Additionally, the activation of neural receptor genes, including acetylcholine receptors, further underlines its intricate pathogenesis involving neurogenic processes. When determining genetic mutations that could possibly contribute to or even predict CNS involvement, Pemmaraju et al. demonstrated in their study that BPDCN patients who were found to have CNS involvement had an increased frequency of *TET2* mutations or alterations [18,26].

## 3. Diagnosing BPDCN with CNS Involvement

With such a rare incidence, diagnosing BPDCN has posed a unique challenge to clinicians. However, guidelines recently set by the North American Blastic Plasmacytoid Dendritic Cell Neoplasm Consortium in 2023 aimed to and successfully outlined definite guidelines for diagnosing and ordering an appropriate workup for patients with BPCDN. For patients suspected to have BPDCN, an initial workup includes routine laboratory studies (complete blood count with differential, chemistry panel, lactate dehydrogenase, liver function tests, coagulation studies, uric acid and peripheral blood smear), bone marrow aspirate with flow cytometry, immunohistochemistry, cytogenics, next-generation sequencing, imaging (positron emission tomography or computed tomography) of lymph nodes and extramedullary disease, dermatology consultations for full skin assessments and potential biopsy, lymph node biopsies, etc. The consortium also recommends using the Modified Severity-Weighted Assessment Tool (mSWAT) to determine the amount of skin involvement [24].

Diagnosing BPDCN with CNS involvement has posed unique challenges due to the fact that lumbar punctures (LPs) have not been a part of standard diagnostic practice for BPDCN, and neither is performing serial LPs. It has been posited that due to the paucity of data and the extremely low incidence of BPDCN and even rarer involvement of the CNS with this malignancy, many diagnostic centers in the past have failed to include LPs as a part of standard diagnostic workup [14,27]. Notably, a majority of patients with CNS involvement remain asymptomatic. In a study by Martín-Martín et al., 60% of patients with BPDCN tested positive for CNS involvement and reported being asymptomatic. Moreover, several studies have indicated that patients with relapse of the disease (33–100%) were shown to have CNS involvement at the time [3,27,28].

However, despite the current challenge to diagnose CNS involvement, the North American Blastic Plasmacytoid Dendritic Cell Neoplasm Consortium has made steps to address this issue. Apart from emphasizing the multidisciplinary focus, with close collaboration between hematologist-oncologists, dermatologists, and pathologists, on the diagnostic workup for initial diagnosis of the disease as outlined above, the protocol notably includes conducting LPs to study for cerebral spinal fluid (CSF) positive for BPDCN cells [24].

Interestingly, observing CSF positive for the disease cells may not be the only way to diagnose CNS involvement for patients with BPDCN. A retrospective study conducted by Davis et al. included patients who had imaging that looked highly suspicious for CNS spread of the disease [20]. Despite being an unorthodox method of categorizing patients with CNS involvement, such methods of inclusion criteria may help patients in which LPs might be inconclusive.

## 4. Treatments for BPDCN with CNS Involvement

**Figure 1:**
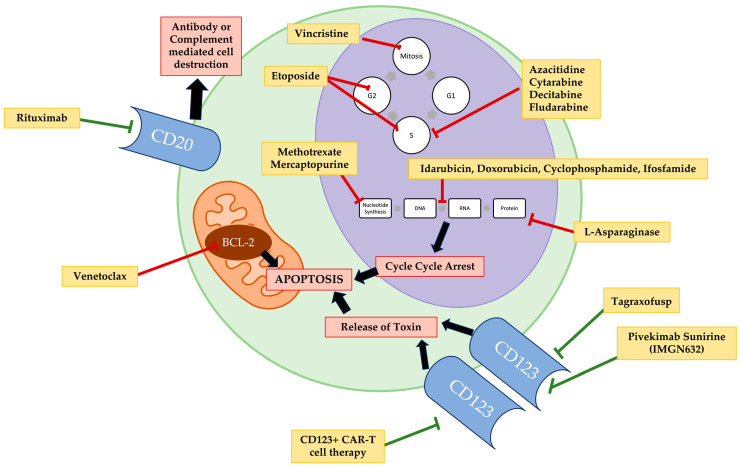
The various targets for therapeutic agents used in treatment regimens for central nervous system involved blastic plasmacytoid dendritic cell neoplasm therapy.

The treatments for BPDCN with CNS involvement resemble the treatments for systemic BPDCN involvement with most targeting intrinsic BPDCN cellular processes or cell surface markers highly expressed on BPDCN (Figure 1).

### 4.1. Intrathecal Therapy (IT)

#### 4.1.1. Methotrexate and Cytarabine IT

Dating back to the 1960s and 70s, intrathecal therapy was a novel way for clinicians to combat leptomeningeal spread of leukemias and lymphomas [29,30,31,32]. Prior to administration of agents directly to the CSF, patients either underwent brain irradiation or multiple LPs for administration of medication, which was not ideal. Medication administered through LPs were inefficient, incorrectly distributed, or had local and systemic toxicities that were not well tolerated by patients [33]. After determining specific formulations and techniques for therapy administration, a series of case studies first identified methotrexate, an antimetabolite which acts as a folate antagonist preventing synthesis of purines and pyrimidines, hence halting DNA synthesis, as one of the first agents which could be effective in treating patients with the spread of leukemia to the CSF. Shortly after, cytarabine, a nucleoside analogue to cytidine which disrupts the DNA synthesis process, was determined to be an additional intrathecal agent which could contribute to reversal of CSF leukemia disease [30,32]. Clinicians noted that intrathecal therapy allowed for a high degree of penetration into the CNS and maintained optimal drug concentrations of either drug [33]. Historically, the standard therapy that had been administered to patients with BPDCN was with systemic ALL or AML chemotherapy. Given the possibility of CNS spread of the disease, including CNS IT in a treatment protocol as a form of CNS prophylaxis for these patients naturally followed. Ultimately, the combination or single use of either methotrexate or cytarabine in IT therapy has proven to help treat CSF+ BPDCN and is used as a prophylaxis for preventing disease spread to the CNS. A retrospective study by Davis et al. involving 11 patients from multiple centers highlighted the efficacy of IT methotrexate and cytarabine treatments. Of these patients, nine received more than three IT sessions [19,26]. A study by Zhang et al. corroborates these findings, and investigators have implemented protocol of alternating methotrexate and cytarabine IT for patients, with ongoing assessment of its efficacy [34].

#### 4.1.2. IT + Tagraxofusp

Tagraxofusp (TAG) is a novel therapy specifically targeting CD123, a surface protein found on BPDCN cells [26,31]. It is a recombinant fusion protein combining interleukin-3 (IL-3) with diphtheria toxin. After the fusion protein is endocytosed into the cell, the diphtheria toxin travels to the cytoplasm after being cleaved into its active form. The toxin adheres to and inhibits Elongation Factor-2 (EF-2), which is responsible for translocation of ribosomes along the mRNA, thus effectively halting protein synthesis and triggering cell apoptosis [35,36]. Approved by the Food and Drug Administration (FDA) for treatment of BPDCN in 2018, the treatment was used for systemic therapy [37]. However, its effect on CNS symptoms of BPDCN was not investigated as patients with CNS symptoms were excluded from the groundbreaking trial conducted by Pemmaraju et al. The study notes that none of the 11 patients’ treatment with TAG had a CNS relapse of the disease in the study; however, patients were not assessed for CNS involvement with LPs throughout the duration of the study, and patients with known CNS disease were excluded from the study. Recently, it has been seen that patients who did not receive CNS prophylaxis at the time of treatment with TAG have reported CNS symptoms at the time of relapse. As seen in the retrospective study by Davis et al., two patients who received TAG as a first-line treatment and fewer than two IT treatments had poor outcomes (0.5 and 0.1 months from the time of CNS involvement) [20]. Rivoli et al. explored the coadministration of methotrexate/cytarabine intrathecal therapy with tagraxofusp and reported remission in three out of five patients with CNS positive disease, with no reported relapses [27]. Despite being a promising systemic therapeutic for treating BPDCN, TAG has been known to cause capillary leak syndrome (CLS) in patients. Initially seen through a drop in albumin, TAG has been known to cause a spectrum of Grade I to Grade V (death) CLS adverse effect in 18% patients in the study conducted by Pemmaraju et al. Other adverse effects of TAG therapy (either the 12 µg or 7 µg dose) involved hepatic dysfunction and thrombocytopenia [37].

#### 4.1.3. IT + Venetoclax

Venetoclax, a B Cell Lymphoma-2 (BLC-2) inhibitor approved for treating chronic lymphocytic leukemia (CLL) is under investigation for BPDCN treatment. A gene expression analysis by Sapienza et al. demonstrated that BCL-2, an antiapoptotic protein, was readily expressed in abnormal pDC cells found in BPDCN [25]. This was further explored by Montero et al. when immunohistochemistry on BPDCN biopsies from skin and bone marrow all demonstrated prominent BCL-2 staining. When looking at targeting the BLC-2 dependency that BPDCN cells have with Venetoclax, Montero et al. first studied two patients with BPDCN that had spread to bone marrow and lymph nodes and had diffuse cutaneous involvement who were treated with increasing weekly doses of venetoclax. Both patients had a remarkable cutaneous response, with a decreased cutaneous disease burden for both patients. The second patients multimodal disease regressed and blast count decreased by 41%. He remained on Venetoclax treatment, however, after 12 weeks the disease had progressed [38]. A study by Albiol et al. showcased disease progression in a relapsed patient with CNS and liver involvement treated with IT therapy and Venetoclax. The patient had previously undergone an AML-like treatment regime followed by allo hematopoietic stem cell transplantation (HSCT) [39].

### 4.2. AML-Type and ALL-Type Therapeutics

Prior to the 2018 approval of Taxgraxofusp, a first-line treatment for BPDCN involved treating patients with AML-type therapies such as anthracycline plus cytarabine; mitoxantrone, ifosfamide, cytarabine, and etoposide (MICE); ifosfamide, cytarabine, and etoposide (ICE); cyclophosphamide, doxorubicin, vincristine, and prednisone (CHOP); cyclophosphamide, doxorubicin, vincristine, etoposide, and prednisone (CHOEP); fludarabine, cytarabine, and granulocyte colony-stimulating factor (G-CSF) (FLAG); or FLAG and idarubicin (FLAG-IDA). Less commonly, ALL-Type or lymphoma therapies were used such as hyperfractionated cyclophosphamide, vincristine, doxorubicin, and dexamethasone (HyperCVAD); 6-mercaptopurine, vincristine, methotrexate, prednisolone (POMP); daunorubicin, vincristine, l-asparaginase, prednisolone, methotrexate, cyclophosphamide, cytarabine, 6-mercaptopurine (MRC UKALL XII) or the Gruppo Italiano Malattie Ematologiche dell’Adulto (GIMEMA) ALL trial therapy [12].

The overall survival (OS) using such treatments is quite poor as relapsed BPDCN has been found to be resistant to chemotherapeutics [40,41]. Although complete response (CR) can commonly be achieved with AML/ALL-type systemic therapy, they are short-lived and relapse is commonly present with CNS symptoms. Thus indicating that systemic therapeutics may not be the most effective in halting or combating the spread of the disease to the CNS despite including IT treatment in some ALL regimens. In a trial conducted by Feuillard et al., 23 patients were given different chemotherapeutic agents (either AML or ALL regimens). CR was achieved in 18 patients (78%). However, of the 18 patients who achieved CR, 15 (83%) had a relapse within 3–18 months [3]. This was additionally supported by a study conducted by Pemmaraju et al. which demonstrated that with 13 patients receiving HyperCVAD, CHOP, and oral methotrexate, 10 patients achieved CR (76.9%) with a median OS between 29 months (1–44 months) [42].

Between AML-type therapy and ALL-therapy, several trials conducted on patients with BPDCN from several centers have indicated that the latter may prove to be the more effective therapeutic regime for patients with BPDCN. The Hellenic group compared whether the AML or ALL treatments amongst patients with BPDCN. Of the 19 patients given chemotherapy, 9 were treated with ALL-type therapy (cytarabine, fludarabine, idarubicin, vincristine, etoposide, mitoxantrone, prednisolone and dexamethasone combination therapy, CHOP, and CHOEP) and 6 with AML-type therapy (HyperCVAD, MRC UKALL XII, and POMP). All 9 patients who received ALL-type therapy achieved CR (100%), while 3 patients who received AML-type therapy achieved CR (50%). Out of the 9 patients who achieved CR after ALL-type therapy, 3 patients relapsed (33.3%), while 1 out of the 3 patients who achieved CR after AML-type therapy relapsed (33.3%). The study proposed that ALL-type regimens were a superior form of systemic chemotherapeutic when compared to AML-type regimens [9]. Another study, which also indicated ALL-type therapy as having improved outcomes for patients, was conducted by Martín-Martín et al., in which 7 patients were treated with ALL-type therapy, 9 patients were treated with AML-type therapy, and 9 patients were treated with C(H)OP-type therapy. All patients belonging to ALL and AML treatment groups achieved CR, while 7 patients in the C(H)OP-type therapy achieved CR (78%). With regard to relapse, 3 patients who received ALL-type therapy relapsed (43%), 7 patients who received AML-type therapy relapsed (78%), and all 7 patients who received C(H)OP-type therapy relapsed (100%). Based on comparisons conducted in the study with regard to relapse percentages, ALL-type therapy versus C(H)OP therapy proved to be significant (*p* = 0.04) [6].

Yet, consistency of trends between these trials regarding which treatment may prove to be the most effective for patients with BPDCN still varies. One of the largest investigations into induction chemotherapy was conducted by Pagano et al. in 2013, where 26 patients were treated with AML-type therapy (MICE, ICE, FLAG and FLAG-IDA) and 15 with ALL/lymphoma-type therapy (HyperCVAD, GIMEMA ALL trial therapy, CHOP, and CHOEP), with 7 patients and 10 patients achieving CR, respectively. Contrary to what has been seen in previous studies, none of the patients treated with AML-type therapy relapsed, while 6 patients (60%) treated with ALL-type therapy relapsed [2].

While many studies mention having BPDCN patients having undergone ALL/AML-type treatment presenting with CNS involvement at the time of relapse, few have documented the exact numbers of CNS relapses in each type of treatment group. Pagano et al. demonstrated that out of 6 relapsed patients, 3 patients (50%) presented with CNS involved BPDCN at the time of relapse. Two of the individuals had previously undergone AML-type therapies, while one was treated with an ALL-Lymphoma-type regimen. Notably, none of these patients had received prior intrathecal prophylaxis [2]. Meanwhile, the study by Feuillard et al. also showed that despite bone marrow infiltration being the most commonly presented manifestation of relapse in patients with BPDCN, 33% (5 out of 15 patients) presented with CNS involvement at the time of relapse [3]. However, Martín-Martín et al. showed that out of 23 patients who relapsed after undergoing AML, ALL, or C(H)OP-type therapy, 6 presented with CNS involvement or CNS progression (26.1%). Of note, patients with ALL- and AML-type therapy had the lowest percentages of CNS relapse or progression (17% and 25%, respectively), while patients treated with C(H)OP therapy had the highest percentage of CNS relapse or progression (83%) [6].

However, other studies have shown that either AML- or ALL-type treatments in combination with other types of therapeutics may be beneficial for patients. He et al. presented a case where HyperCVAD and venetoclax-based therapy for a pediatric patient with CNS, orbital, and skin involvement resulted in complete remission. The individual remained disease-free for over 200 days post-receiving an allogeneic hematopoietic stem cell transplant, highlighting the potential effectiveness of this combination therapy in multifocal disease control [43]. Additionally, Davis et al. documented promising outcomes in two patients undergoing HyperCVAD therapy along with methotrexate and cytarabine (MA) intrathecal combination therapy (HyperCVAD/MA). These individuals showcased a complete CNS response, with overall survival times from BPDCN diagnosis reaching 50.3 and 10.8 months, respectively [20].

#### Post-AML/ALL-Type Therapy Hematopoietic Stem Cell Transplant (HSCT)

While intensive chemotherapy followed by HSCT may prove to be curative for patients, it has only been effective in a minority of patients. In two of the largest known studies conducted with BPDCN patients receiving allogenic HSCT (allo-HSCT), after AML, ALL, or Lymphoma-type therapy, 32% to 35.7% of patients were shown to have disease relapse [44,45]. With regard to autologous HSCT, with patients also most commonly being treated with AML, ALL, or Lymphoma-type therapy, results have shown a relapse of disease in 18.1% of BPDCN patients [44]. Interestingly, while numbers of patient relapses are detailed, there is lack of documentation regarding the primary manifestation of disease in these patient populations. As the disease is known to occur in elderly individuals, tolerating such rigorous therapy in addition to being eligible for stem cell transplantation is another challenge for the larger majority of patients with CNS-involved BPDCN.

### 4.3. Emerging Therapeutics for BPDCN

Despite limited options for clinicians to choose from when it comes to standard treatment, many clinical trials are currently being conducted which explore novel treatments for BPDCN [18,23]. These trials include novel therapeutic agents such as chimeric antigen receptor T cell (CAR T) therapy and monoclonal antibody therapy in the form of IMGN632 (a CD123-specific humanized IgG monoclonal antibody). While there might not be targeted trials focusing on CNS involvement specifically, some trials have included patients with CNS involvement at the time of diagnosis or with a relapse as summarized in Table 1 [46].

A Phase I clinical trial using venetoclax (NCT03485547) in the treatment of any adults (≥18 years old) diagnosed with BPDCN has not provided any exclusion criteria for a patient presenting with CNS involvement at the time of diagnosis or relapsed disease [47].

Patients with CNS leukemic involvement refractory to IT therapy, after undergoing lymphodepletion, will be eligible to be enrolled in arm two of the Phase I trial, which is currently utilizing CD123+ CAR T cells to treat patients with persistent or recurrent BPDCN after first-line therapy (NCT02159495) [48]. CAR T cell therapy, first developed in the 1980s, works by the creation of a CAR consisting of three domains. The first domain, known as the single-chain variable fragment (scFv) is responsible for the identification of a specific target, in this case, CD123+ cells. The second domain is derived from T cell signaling molecules in order to allow for intercellular signaling, allowing for recruitment of more immune cells to destroy malignant BPDCN cells independent of the major histocompatibility complex. The final domain consists of the transmembrane region which connects the first and second domain as well as maintains ideal conformation needed for the CAR to work [36].

A Phase II trial currently enrolling patients with BPDCN using multidrug chemotherapy (NCT03599960) includes treatment for patients with CNS involvement. The trial consists of treating patients with three cycles (21 days) of idarubicin, methotrexate, L-asparaginase, and dexamethasone followed by either autologous or allogenic stem cell transplant (SCT), or those not eligible will receive 28 day cycles of methotrexate, L-asparaginase, and dexamethasone [49].

A Phase II trial using venetoclax and decitabine (NCT03404193) in treating participants with relapsed/refractory acute myeloid leukemia or relapsed high-risk myelodysplastic syndrome (BPDCN is included) allows for CNS prophylaxis with IT consisting of oral hydroxyurea and/or one dose of cytarabine. Patients with controlled CNS disease are included in the study as well. However, patients with symptomatic CNS leukemia or patients with poorly controlled CNS leukemia are excluded in the study [50].

## 5. Conclusions and Future Directions

Over the years, due to better understanding of BPDCN and the biology of the disease, therapeutics have evolved to help with increasing the overall survival for these patients. However, due to the rarer incidence of spread of disease to the CNS, the diagnosis and treatment of these subsets of patients are still evolving. Prophylactic LPs are not standard practice for monitoring BPDCN patients, further complicating the tracking of CNS involvement. The nuances of asymptomatic versus symptomatic CNS involvement in BPDCN underscore the need for more comprehensive diagnostic approaches and consideration of CNS manifestations in both diagnosis and treatment protocols. Groundbreaking studies conducted by Pemmaraju et al. for Tagraxofusp treatment and Montero et al. for Venetoclax as a BPDCN therapy excluded patients with CNS presentations, thus limiting comprehensive understanding and tailored treatment strategies for this subset of patients. In addition to this, documentation of the primary manifestation of relapse in patients after receiving HSCT is important. Not only will this help elucidate whether HSCT may prove to be curative for CNS disease in BPDCN but may further explain the pathophysiology of the disease with regard to its CNS involved BPDCN. Clinical trials are critical for exploration of future treatment options for patients with BPDCN in its various manifestations. Yet, progress with regard to CNS treatment options may prove to be elusive when therapies such as IMGN632, which has received breakthrough designation therapy from the US Food and Drug Administration, have exclusion criteria for patients with known CNS disease. Currently, IT therapy is the only specific therapeutic with respect to treating CNS disease in BPDCN patients, showing that this is a major unmet area of drug development. There are additional promising clinical trials which can hopefully elucidate more or provide clinicians with additional treatment alternatives when it comes to BPDCN patients having CNS involvement.

## Figures and Tables

**Table 1:** A list of current clinical trials, disease status of patients being enrolled, and central nervous system exclusion criteria for patients diagnosed with blastic plasmacytoid dendritic cell neoplasm.

Trial Identifier	Agents	Eligible Patients	Exclusion CNS Criteria
NCT03485547	Venetoclax	BPDCN	None
NCT03113643	TAG + Azacitidine/Azacitidine and Venetoclax	Relapsed/refractory BPDCN	Yes
NCT02159495	Autologous/Allogeneic CD123CAR-CD28-CD3zeta-EGFRt-expressing T Lymphocytes	Relapsed/refractory BPDCN after first-line therapy	None—Lymphodepletion for IT
NCT04230265	UniCAR02-T Cells	BPDCN	Yes
NCT03386513	IMGN632	BPDCN	Yes
NCT04109482	MB-102	Relapsed/Refractory BPDCN	Yes
NCT04317781	TAG	BPDCN post-HSCT	Yes
NCT04216524	Venetoclax + TAG + Cyclophosphamide + Cytarabine + Doxorubicin + Mercaptopurine + Methotrexate + Rituximab + Vincristine	BPDCN	Yes
NCT03599960	Idarubicin + Methotrexate + L-asparaginase + Dexamethasone followed by allo- or auto-SCT or Methotrexate + L-asparaginase + Dexamethasone	BPDCN	None
NCT03404193	Venetoclax + Decitabine	BPDCN	Partial
